# Poverty reduction and equity benefits of introducing or scaling up measles, rotavirus and pneumococcal vaccines in low-income and middle-income countries: a modelling study

**DOI:** 10.1136/bmjgh-2017-000613

**Published:** 2018-04-09

**Authors:** Carlos Riumallo-Herl, Angela Y Chang, Samantha Clark, Dagna Constenla, Andrew Clark, Logan Brenzel, Stéphane Verguet

**Affiliations:** 1 Department of Global Health and Population, Harvard TH Chan School of Public Health, Boston, Massachusetts, USA; 2 Department of Applied Economics, Erasmus School of Economics, Erasmus University of Rotterdam, Rotterdam, The Netherlands; 3 Pharmaceutical Outcomes Research and Policy Program, University of Washington, Seattle, Washington, USA; 4 Department of International Health, Johns Hopkins Bloomberg School of Public Health, Baltimore, Maryland, USA; 5 Department of Health Services Research and Policy, London School of Hygiene and Tropical Medicine, London, UK; 6 Bill and Melinda Gates Foundation, Washington DC, USA

**Keywords:** vaccines, measles, rotavirus, pneumococcal pneumonia, out-of-pocket costs, catastrophic health costs, financial risk protection, impoverishment, poverty, equity

## Abstract

**Introduction:**

Beyond their impact on health, vaccines can lead to large economic benefits. While most economic evaluations of vaccines have focused on the health impact of vaccines at a national scale, it is critical to understand how their impact is distributed along population subgroups.

**Methods:**

We build a financial risk protection model to evaluate the impact of immunisation against measles, severe pneumococcal disease and severe rotavirus for birth cohorts vaccinated over 2016–2030 for three scenarios in 41 Gavi-eligible countries: no immunisation, current immunisation coverage forecasts and the current immunisation coverage enhanced with funding support. We distribute modelled disease cases per socioeconomic group and derive the number of cases of: (1) catastrophic health costs (CHCs) and (2) medical impoverishment.

**Results:**

In the absence of any vaccine coverage, the number of CHC cases attributable to measles, severe pneumococcal disease and severe rotavirus would be approximately 18.9 million, 6.6 million and 2.2 million, respectively. Expanding vaccine coverage would reduce this number by up to 90%, 30% and 40% in each case. More importantly, we find a higher share of CHC incidence among the poorest quintiles who consequently benefit more from vaccine expansion.

**Conclusion:**

Our findings contribute to the understanding of how vaccines can have a broad economic impact. In particular, we find that immunisation programmes can reduce the proportion of households facing catastrophic payments from out-of-pocket health expenses, mainly in lower socioeconomic groups. Thus, vaccines could have an important role in poverty reduction.

**Key messages**

**What is already known about this topic?**

- Vaccines have a large beneficial impact beyond health and provide important returns on investments.
- Scale up of vaccines could have a large role in achieving the Sustainable Development Goals and reducing poverty.

**What are the new findings?**

- We develop a methodology that evaluates the financial risk protection (FRP) benefits of vaccines across income quintiles, thus adding equity and distributional components to previous evaluations.
- The FRP benefits of vaccines are mostly accrued by households in the lowest income quintiles, thus emphasising the role of vaccines as a pro-poor intervention.

**Recommendations for policy**

- Including equity components into economic evaluations will allow policy makers to opt for interventions that target specifically the most vulnerable populations.
- Comparisons across interventions can now be done by integrating fairness arguments by which groups they impact more.
- Vaccines represent a valuable pro-poor intervention that not only improves health but also protects poor households from catastrophic and impoverishing health expenditures.

## Introduction

In September 2015, leaders across the world adopted the 17 Sustainable Development Goals (SDGs) that will guide international development policy through 2030.^1^ These goals build on the Millennium Development Goals and maintain eliminating poverty as one of the overarching goals in the decades to come, for example, SDG1 of ‘ending poverty in all its forms everywhere’.^1^ According to current estimates, approximately 800 million people still live in extreme poverty, and one in five individuals in developing regions is considered extreme poor, that is, living on less than $1.90 (2011 Purchasing Power Parity, PPP) a day.^2^ Furthermore, it was estimated that over 150 million people annually suffer from catastrophic costs when paying for their family’s healthcare between 1990 and 2003.^3^ These estimates and current population growth in these regions emphasise the magnitude of the challenge of eradicating poverty by 2030 represents. Furthermore, this highlights the need for evidence-based policy making that identifies areas where larger benefits can be attained.

One area that has gained impetus as a cause of poverty and financial hardship is healthcare expenditure. A number of studies have identified that out-of-pocket (OOP) healthcare payments are important predictors of financial hardship and poverty.^3–6^ The importance of healthcare payments as a cause of financial hardship was acknowledged by world leaders when establishing financial risk protection (FRP) as one of the targets within the goal of universal health coverage (UHC) including measures against catastrophic health expenditures and medical impoverishment due to health.^7–9^ In particular, this is true for lower-income households that are often at greater risk from experiencing catastrophic health expenditures—health expenditures surpassing a certain threshold of total consumption expenditures or income.^10^ This not only highlights the role of healthcare payments in increasing catastrophic health costs (CHCs) but also generates an explicit link between the goals of eradicating poverty and UHC. Consequently, there is a need to identify both health and non-health interventions that can ultimately contribute to reducing healthcare expenditure (increasing FRP) and the incidence of poverty.

One set of health interventions with particularly great potential is vaccination due to its preventive nature and usually lower cost compared with treatment. In May 2012, the World Health Organization (WHO) Global Vaccine Action Plan highlighted vaccinations as essential tools for improving individual-level health globally.^11^ The widespread attention that vaccines have received during the last decade, such as the public endorsement from WHO member states of the Global Vaccine Action Plan, highlights the role that vaccines can play in the broader development agenda. However, despite overwhelming evidence on their efficacy and health impact, the rather limited evidence on their broader distributional economic benefits may constrain their expansion in low-income and middle-income countries that see some vaccines as relatively expensive, especially when many governments are resource-constrained.

Studies that have evaluated the economic benefits of vaccines have found overall large effects.^12^ In particular, a recent analysis found that immunisation programmes could yield substantial financial returns that range from 10 to 25 times that which was invested.^13^ One limitation was, however, the focus on aggregate economic benefits and little discussion on the distributional impact (eg, per socioeconomic group) of vaccines. Current evidence from the literature has shown that morbidity and mortality of vaccine-preventable diseases are disproportionally reduced among the poor when vaccination programmes are implemented.^14–16^ This is mirrored at the international level where vaccine-preventable diseases have a higher impact on low-income and middle-income countries.^17^ The combination of both the within and between country evidence hints that vaccines could have important distributional economic benefits, and that therefore, expanding vaccine coverage could contribute to poverty reduction.^12 18^

As the international community seeks to achieve the SDGs, vaccines may be able to contribute substantially to the post-2015 poverty reduction agenda. This paper develops a cost-epidemiological simulation model to estimate the distributional FRP benefits of three vaccines (measles, rotavirus and pneumococcal conjugate), in low-income and middle-income countries.

## Methods

In this paper, we develop methods to estimate the effect of three vaccines–measles, rotavirus and pneumococcal conjugate vaccine–on FRP in low-income and middle-income countries. We then illustrate these methods and apply them to the estimation of the number of cases of (1) medical impoverishment and (2) CHCs in 41 countries eligible for funding from Gavi, the Vaccine Alliance (online supplementary appendix table A1) for birth cohorts born over the time period 2016–2030. We restricted our analysis to a subset of the total Gavi-eligible countries for which Demographic and Health Survey (DHS) data were available after 2010 to develop a distributional analysis by socioeconomic group (ie, income quintile). With regard to medical impoverishment, a poverty event is counted when household income minus healthcare costs falls below the World Bank poverty line of $1.90 per day—assuming household income was previously above the poverty line.^19^ Alternatively, a case of CHC is counted when OOP healthcare costs are larger than 20% of household income. These measures of household economic well-being are routinely used by WHO and World Bank to evaluate the financial impact of health policies on households.^2 3 20^

10.1136/bmjgh-2017-000613.supp1 Supplementary file 1

We estimate these two metrics at the monthly level for three immunisation coverage scenarios: (1) no immunisation, (2) the current forecasted immunisation programmes, that is, the current trends of immunisation coverage without any additional funding (‘current trends’) and (3) the current trends of immunisation coverage enhanced with Gavi funding support for the expansion or implementation of new vaccines (‘best case’). These scenarios rely on the model predictions developed by a series of experts, including Gavi, on the long-term volume of vaccines likely to be required among Gavi-eligible countries in the future.^21^ Online supplementary appendix figure A1 presents the average coverage rate of each vaccine for scenarios 2 and 3 in cohorts born between 2000 and 2030. For each scenario, we use a simulation model to obtain aggregate estimates of cases of poverty and CHC over 2016–2030 across income quintiles in each country. Per quintile, FRP is calculated as the difference in terms of cases of either poverty or CHC between either scenario (2) or (3) and the base case (1).

### Modelling approach

The FRP model can be described in four steps. First, we obtain the number of incident cases for each disease from disease-specific models (measles, rotavirus and pneumococcal disease) by country. For rotavirus, the cases considered only include severe rotavirus defined by moderate or severe dehydration attributable to rotavirus.^22^ For pneumococcal disease, cases considered included severe pneumococcal pneumonia, meningitis and non-pneumonia/non-meningitis excluding non-severe pneumonia, otitis media and meningitis sequelae.^23^ Severe pneumococcal pneumonia was defined according to WHO definition of coughing and difficulty breathing.^24^ Further detail on the disease models that provide the disease case inputs for this study is described elsewhere.^23 25 26^ These incident cases are then distributed across income quintiles in each country population. For this, we use a distributional risk approach that varies by specific disease (measles, severe rotavirus or severe pneumococcal disease) developed by Chang *et al*.^27^ This approach uses the prevalence and relative risk of a set of risk factors as well as the vaccination coverage gradient to distribute the number of disease cases across income quintiles. Finally, using DHS data, we derive ‘healthcare utilisation’ likelihoods, that is, the differential probability in healthcare utilisation across income quintiles, to estimate the disease cases treated in each income quintile. Due to the paucity of data on disease incidence within a household, we assumed only one disease case per household.

In step 2, we estimate the total health-related costs incurred by households that can be attributed to each treated disease case. We include OOP direct medical costs: treatment costs for the disease, transportation costs as well as indirect costs for caretaking due to time losses. For this purpose, we use estimates of country treatment provider costs,^2 28–42^ the average hospitalisation length per disease, the proportion of cases that are hospitalised and transportation costs from/to the health facility.^42^ Additionally, we consider indirect costs defined as the product of hospitalisation or outpatient duration with the hourly wage data for each country.^34 43^ The direct medical costs represent the total cost for the health system; therefore, we use the share of OOP expenditures to total health expenditures^2^ as a proxy for the fraction of the costs that would be borne by individuals. With this data, we estimate total expected (correcting for utilisation) healthcare expenditures for each disease. All costs figures are expressed in $ 2011 PPP in order to follow the World Bank standard of poverty measurement.^2^

In the third step, we define the CHC and poverty cases. For that, we count a CHC case when total health costs exceed 20% of the monthly household income per capita^44^ and a case of poverty when monthly household income per capita minus health costs becomes lower than the $1.90 2011 PPP per day poverty line. Monthly household income is drawn from a simulated gamma distribution whose shape and scale parameters are based on the country’s gross domestic product per capita and Gini coefficient.^45 46^

Finally, we aggregate the number of cases (either CHC or poverty), annually and cumulatively over 2016–2030, for all 41 countries. Thus, per scenario and per disease, we obtain numbers of cases of CHC and poverty accumulated in this period across income quintiles. All analyses of FRP (CHC and poverty) were pursued using the R Studio V.1.0.143 (www.r-project.org).

### Input data

Table 1 lists all the inputs used in the simulation model, including the average number of annual disease cases in all countries under the three different coverage scenarios over 2016–2030 (no vaccination, current trends and best case), the average patient cost across all countries that an individual would face with each disease and disease incidence and utilisation gradients.

**Table 1:** Inputs used in the simulation model estimating cases of poverty and catastrophic health costs due to measles, severe rotavirus and severe pneumococcal disease, in 41 low-income and middle-income countries

	Average (min–max) across countries	Reference
(a) Total number of cases (2016–2030) in 1000s		
Scenario 1: no vaccination		
Measles	16 709 (378–107 443)	21 25 26
Severe pneumococcal disease	675 (6–4648)	21 23 25
Severe rotavirus	1555 (28–10 573)	21 23 25
Scenario 2: current coverage trends		
Measles	2196 (8–34 182)	21 25 26
Severe pneumococcal disease	658 (5–4256)	21 23 25
Severe rotavirus	1485 (13–10 573)	21 23 25
Scenario 3: best case		
Measles	1279 (6–14 530)	21 25 26
Severe pneumococcal disease	467 (4–3383)	21 23 25
Severe rotavirus	948 (4–7890)	21 23 25
(b) Provider treatment costs		
Inpatient costs		
Measles	$12.0 (1.4–53.5)	2 30 38–41
Severe pneumococcal disease	$51.2 (6.2–241.3)	2 28–34 37 39–41
Severe rotavirus	$38.0 (4.4–171.0)	2 30 35 36 39–41
Outpatient hospital costs		
Measles	$2.8 (0.6–9.1)	2 30 38–41
Severe pneumococcal disease	$2.7 (0.6–9.1)	2 28–34 37 39–41
Severe rotavirus	$2.7 (0.6–9.1)	2 30 35 36 39–41
Outpatient health centre costs		
Measles	$1.4 (0.3–4.5)	2 30 38–41
Severe pneumococcal disease	$1.3 (0.3–4.5)	2 28–34 37 39–41
Severe rotavirus	$1.3 (0.3–4.5)	2 30 35 36 39–41
Transport costs		
Measles	$2.0 (0.2–9.4)	34
Severe pneumococcal disease	$2.0 (0.2–9.4)	34
Severe rotavirus	$2.1 (0.2–9.4)	34
(c) Health gradients		
Disease case distribution		
1st quintile (poorest)	22% (6–31)	27
2nd quintile	22% (13–25)	27
3rd quintile	20% (15–24)	27
4th quintile	20% (14–30)	27
5th quintile (highest)	16% (11–28)	27
*Healthcare access likelihood*		
1st quintile (poorest)	43% (11–76)	41
2nd quintile	45% (24–76)	41
3rd quintile	49% (25–78)	41
4th quintile	50% (25–75)	41
5th quintile (highest)	56% (33–78)	41

Note. Table presents the average value as well as a minimum and maximum in parentheses for the set of countries studied. Current coverage trends represent current vaccination forecasts. Best case includes additional Gavi funding for the expansion or implementation of vaccines.

### Sensitivity analysis

First, we conduct two univariate sensitivity analyses. For CHC, we vary the threshold from 10% to 40% of household income per capita, instead of 20% (base case). The second univariate analysis evaluates the role of healthcare utilisation and obtains estimates of CHC and poverty cases assuming that all individuals in the country have equivalent utilisation to those of the highest income group. Finally, we conduct a probabilistic sensitivity analysis using Monte Carlo simulations (n=1000 trials), where case distribution, utilisation and costs were varied simultaneously using truncated normal distributions with the inputs’ means and 20% of the means as SD. This allows us to extract the 2.5 and 97.5 percentiles to determine 95% uncertainty ranges (URs) that are incorporated into our results.

## Results

**Figure 1:**
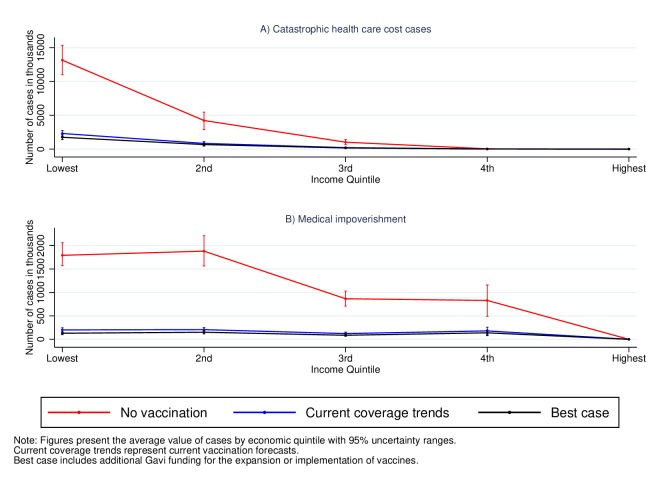
Household cases of catastrophic health costs and medical impoverishment attributable to measles in 41 low-income and middle-income countries for the cohorts born between 2016 and 2030.

Figure 1 presents the number of CHC and medical impoverishment cases attributable to measles for those born from 2016 to 2030 in 41 Gavi-eligible countries by household income quintile. We estimate that approximately 18.9 million households (95% UR 16.4–21.4) would have CHC attributable to measles in the absence of any vaccination coverage. This represents approximately 35% of the birth cohort born in these countries in 2016 assuming one susceptible child per household. The number of CHC decreases to 3.4 million households (2.9–3.4) that is approximately 5% of the 2016 birth cohort in these countries if the current coverage forecasts remain and to 2.6 million (2.2–3.0) if coverage was enhanced with Gavi support. For medical impoverishment, the number of households that would fall under the poverty line due to medical expenditures attributable to measles would be 5.3 million (4.8–5.3) in the absence of any vaccination coverage, 0.7 million (0.6–0.8) under the current trends of immunisation coverage and 0.5 million (0.4–0.6) if Gavi support was provided. Overall, the results show that vaccine coverage can reduce by approximately 90% the incidence of CHC attributable to measles.

**Figure 2:**
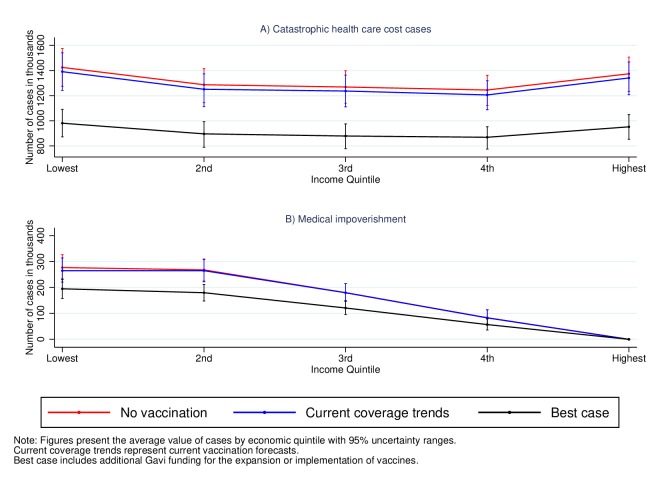
Household cases of catastrophic health costs and medical impoverishment attributable to severe pneumococcal pneumonia in 41 low-income and middle-income countries for the cohorts born between 2016 and 2030.

Figures 2 presents similar estimates attributable to severe pneumococcal disease. We find that approximately 6.6 (6.3–6.9) million households would suffer from CHC between 2016 and 2030 in the absence of vaccine coverage. In contrast to measles, under the current prevailing conditions of vaccine coverage, the number of CHC cases would only decrease slightly to 6.4 million (6.1–6.7) and is explained by the common absence of the pneumococcal conjugate vaccine in current country vaccination programmes. This represents approximately 13% of the 2016 birth cohorts of these countries. This vaccine will be implemented or expanded with Gavi support in some countries, and under this scenario, the number of households with CHC cases would decrease to 4.6 million (4.4–4.8) representing a decrease of approximately 30%. A similar conclusion is drawn from the medical impoverishment estimates where without vaccine coverage, 0.8 million (0.7–0.9) households would fall under the poverty line. This value is similar under the current forecasts, but with Gavi support, the number of households that fall under the poverty line due to healthcare expenditures would decrease to 0.6 million (0.5–0.6).

**Figure 3:**
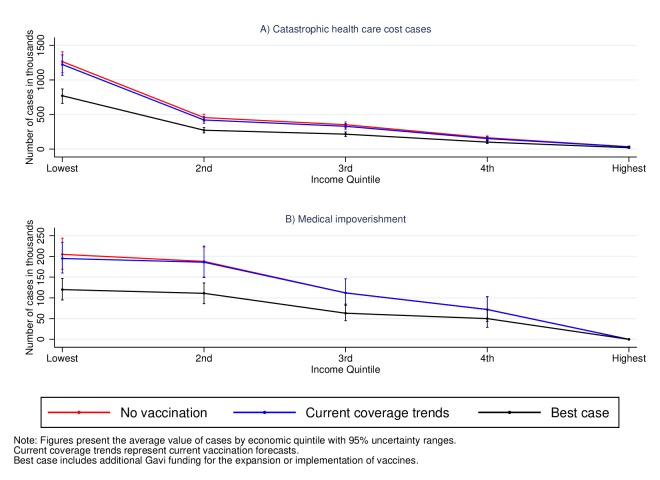
Household cases of catastrophic health costs and medical impoverishment attributable to severe rotavirus in 41 low-income and middle-income countries for the cohorts born between 2016 and 2030.

Figure 3 presents the estimated number of CHC and medical impoverishment cases attributable to severe rotavirus by household income quintile. The numbers are considerably lower than in the cases of measles and severe pneumococcal disease. Without any vaccine coverage, we estimate 2.2 million CHC cases (2.1–2.4) and 0.6 (0.6–0.7) million medical impoverishment cases. These numbers would not significantly decrease under the current forecasts: to 2.1 million (2.0–2.4) and 0.6 million (0.6–0.7), respectively. This is because very few countries have introduced the rotavirus vaccine. Nevertheless, the implementation and expansion of the rotavirus vaccine in the best-case scenario would reduce the number of CHC cases to 1.3 million (1.2–1.5) and medical impoverishment cases to 0.4 (0.3–0.4). As in the case of severe pneumococcal disease, this represents an approximate 40% reduction from the no vaccination or current forecasts scenario.

In the case of measles and severe rotavirus, the figures above also show that the share of CHC and medical impoverishment cases is larger in households from the lower-income quintiles. From the estimates above, we calculate the number of CHC cases averted under the current prevailing conditions with and without Gavi support (table 2) and estimate the share of averted cases in each quintile. Table 2 shows that of the approximately 12.5 million cases of CHC attributable to measles that will be averted under current coverage trends, 75% (95% UR 70–81) of them will occur in the households from the lowest income quintile. The value for severe rotavirus is lower, 40% (30–51), but is nevertheless the largest across income quintiles. This gradient is even smaller for severe pneumococcal disease. However, enhancing the coverage of vaccines to the best-case scenario would not only lead to a larger number of CHC cases averted but also increase the share of protected households in the lowest quintile. For example, with increased Gavi funding, 57% (53–61) of all the averted CHC cases attributable to severe rotavirus would occur in the lowest income quintile. These results highlight that not only can vaccines decrease considerably the number of CHC cases but they also have the potential of disproportionately improving the financial conditions of the poor. The estimates for medical impoverishment lead to similar conclusions with the share of averted cases decreasing with income quintile and emphasising the pro-poor financial benefits of vaccines (online supplementary appendix tables A2 and 3).

**Table 2:** Percentage and number in 1000s of total catastrophic health costs cases averted by vaccines in 41 low-income and middle-income countries for those born between 2016 and 2030

	(1)	(2)	(3)
	Measles vaccine (95% UR)	Pneumococcal conjugate vaccine (95% UR)	Rotavirus vaccine (95% UR)
Current coverage trends			
Lowest	75.2% (69.6–80.8)	22.2% (15.8–28.8)	40.4% (29.6–51.1)
	12 506 (10 636–14 409)	38 (26–51)	47 (31–64)
2nd quintile	19.9% (14.3–25.4)	21.7% (15.2–28.7)	30.3% (19.7–40.7)
	3286 (2290–4268)	37 (24–50)	36 (21–49)
3rd quintile	4.7% (3.1–6.5)	18.4% (12.6–25.3)	19.6% (11.3–27.8)
	779 (501–1045)	32 (20–44)	22 (12–34)
4th quintile	0.1% (0.1–0.2)	20.2% (14.0–26.3)	9.4% (3.9–15.5)
	19 (10–29)	34 (23–46)	11 (4–18)
Highest	0.0% (0.0–0.0)	17.4% (11.6–23.1)	0.0% (0.0–1.6)
	0 (0–1)	30 (18–40)	0 (0–2)
Best case			
Lowest	75.2% (69.7–80.8)	24.7% (22.1–27.2)	57.4% (53.1–61.3)
	13 097 (11 110–15 108)	495 (438–558)	536 (469–604)
2nd quintile	19.9% (14.2–25.3)	20.7% (18.5–22.9)	20.4% (17.6–23.4)
	3442 (2384–4472)	416 (366–465)	190 (161–220)
3rd quintile	4.8% (3.1–6.6)	19.6% (17.3–21.7)	14.8% (12.3–17.5)
	826 (532–1106)	394 (346–442)	138 (114–162)
4th quintile	0.1% (0.1–0.2)	17.7% (15.7–19.6)	6.2% (4.7–7.8)
	20 (11–30)	354 (312–394)	57 (44–72)
Highest	0.0% (0.0–0.0)	17.5% (15.5–19.3)	1.2% (0.6–1.9)
	0 (0–1)	351 (309–391)	10 (6–17)

Note. Current coverage trends represent current vaccination forecasts. Best case includes additional Gavi funding for the expansion or implementation of vaccines. Figures present the average value of cases by economic quintile with 95% URs.

95% URs are given in parentheses.

UR, uncertainty range.

**Figure 4:**
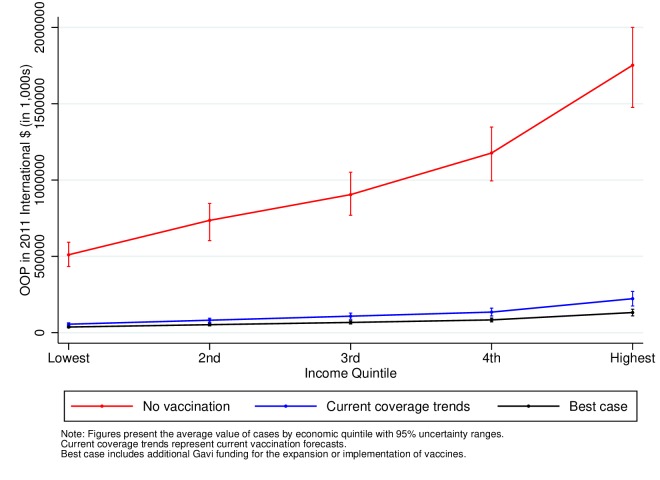
Total OOP health expenditures attributable to measles in 41 low-income and middle-income countries for the cohorts born between 2016 and 2030. OOP, out-of-pocket.

**Figure 5:**
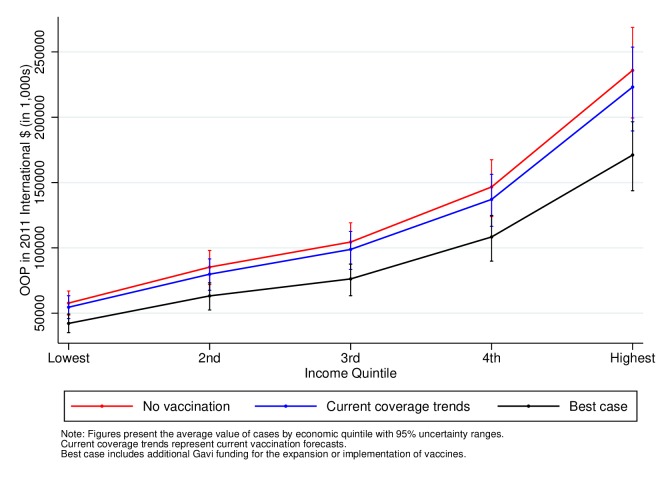
Total OOP health expenditures attributable to severe pneumococcal disease in 41 low-income and middle-income countries for the cohorts born between 2016 and 2030. OOP, out-of-pocket.

**Figure 6:**
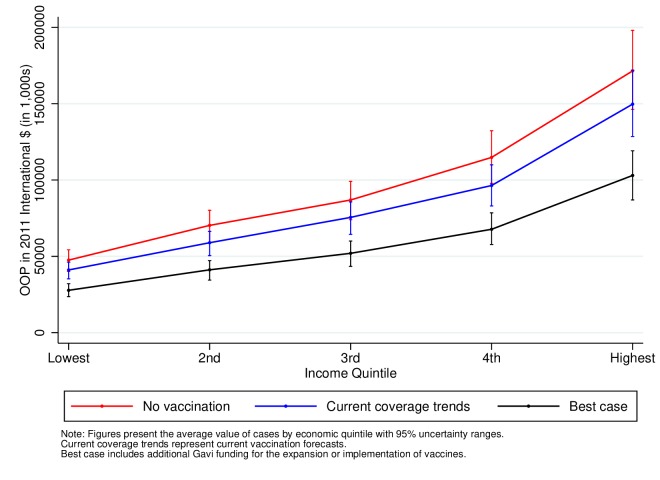
Total OOP health expenditures attributable to severe rotavirus in 41 low-income and middle-income countries for the cohorts born between 2016 and 2030. OOP, out-of-pocket.

Figures 4–6 also show the total OOP expenditures attributable to measles, severe pneumococcal disease and severe rotavirus by income quintile for birth cohorts from 2016 to 2030. In contrast to the results for FRP, expenditures are larger in the highest income quintiles in line with their higher healthcare utilisation. These estimates, however, show that expanding the coverage of vaccines leads to an important reduction in total OOP expenditures. Under the current vaccine coverage forecasts, the amount of OOP expenditures averted would be 4.3 billion (4.1–4.7) 2011 International $ PPP for measles, $36 million (30–43) for severe pneumococcal disease and $70 million (58–82) for severe rotavirus (online supplementary appendix table A4). The averted expenditures further increase to $4.6 billion (4.2–5.0) for measles, $168 million (156–181) for severe pneumococcal disease and $200 million for severe rotavirus (184–215) when including Gavi support (online supplementary appendix table A4). In line with the figures, the larger share of the total OOP health expenditures averted would occur in the higher-income quintiles as shown in online supplementary appendix table A5.

### Sensitivity analysis

Online supplementary appendix tables A2 to 10 present the univariate sensitivity analyses testing for the effects of the CHC threshold, the assumptions underlying the fraction of healthcare utilisation and the distribution of vaccine coverage with and without total fertility rate (TFR) adjustments. In the case of the thresholds (online supplementary appendix figures A2,3 and 4), we find that, as expected, increasing the threshold leads to a lower number of cases. However, the results still show large number of CHC cases and most importantly in the bottom income quintiles. One important aspect to note is that if we used a threshold of 10%, the total number of CHC cases in the absence of immunisation would be 49 million, 7 million and 5 million for measles, severe pneumococcal disease and severe rotavirus, respectively. This would represent approximately 100%, 15% and 10% of the 2016 birth cohort of these 41 countries. Under the best-case scenario, the number of cases using the 10% threshold would decrease to 6 million, 3 million and 3 million, respectively, emphasising the importance of vaccines even when considering different thresholds. Online supplementary appendix figures A5 to 7 present the results assuming that all income quintiles achieve the highest utilisation observed in the country. As expected, the number of CHC and poverty cases increases since individuals are more likely to use healthcare if ill while facing similar OOP costs. The effects are substantial and show an increase under the 20% threshold to approximately 23 million cases for measles, 8 million cases for severe pneumococcal disease and 3 million for severe rotavirus. As before, under the best-case scenario, the numbers decrease to 3 million, 6 million and 2 million, respectively. The similarity of the conclusions emphasises the importance of vaccines and their role in protecting households financially from OOP health expenditures. Finally, online supplementary appendix figures A8 to 10 show that TFR adjustments across quintiles do not lead to important differences in the results.

## Discussion

This paper develops a methodology to estimate how cases of CHC and medical impoverishment incurred by vaccine-preventable diseases would be distributed across socioeconomic status and to quantify the likely FRP benefits of immunisation programmes. We find that in the absence of immunisation, there would be about 19 million CHC cases attributable to measles, 7 million to severe pneumococcal disease and 2 million to severe rotavirus for cohorts born between 2016 and 2030 in 41 low-income and middle-income countries. Conversely, if projected coverage increases to the best-case scenario, that is, including Gavi funding for the expansion and implementation of new vaccines, the incidence of CHC could decrease to 3 million, 5 million and 1 million, respectively. Similar estimates are obtained when considering other indicators of FRP such as cases of medical impoverishment. These results emphasise the fundamental role that vaccines can have in the global agenda to protect households’ financial well-being as well as reduce the incidence of medical impoverishment.

Furthermore, not only the burden of CHC and medical impoverishment would be greater in the lowest income quintiles but also the expanding vaccine coverage could provide larger FRP to the poorest quintiles. An important protective gradient could incur where the share of averted cases in relation to the total number of cases averted would be larger in the lowest income quintiles. Consequently, vaccines not only could enhance financial protection from OOP health expenditures but also could benefit more those that are worst off.

Finally, we estimate that under the best-case scenario, current coverage trends with enhanced Gavi funding, the amount of OOP health expenditures averted would surpass $4.5 billion (2011 International $) attributable to measles, $168 million attributable to severe pneumococcal disease and $200 million attributable to severe rotavirus. Therefore, expanding vaccine coverage would improve household financial well-being by increasing disposable income that could further improve their living conditions.

Overall, this paper provides additional information for decision makers to consider when setting national priorities and can help place poverty reduction as a major outcome of health interventions with the objective of achieving the SDGs by 2030. In particular, this methodology helps incorporating poverty reduction and FRP as a criterion to design a basic health benefits package including publicly financed immunisations, where both FRP and cost-effectiveness are taken into account, consistent with extended cost-effectiveness analysis methods^47–49^ and frameworks of multicriteria decision analysis.^50^ Our approach also quantifies cost-effective health-policy investments in terms of poverty reduction that can be included in discussions outside of the health sector such as agriculture or education and for ministries of finance and development.^51^

Our analysis presents, nevertheless, several limitations. First, data on OOP expenditures and impoverishment are lacking substantially, and therefore, we had to rely on information available for a small set of countries and imputed data.^52–55^ Additionally, there is paucity of information on treatment costs and OOP costs that emphasise the need of further studies in this area. Our CHC and poverty cases estimates rely on disease case outputs from disease and forecast models that have their own limitations.^21 25 26 42^ Furthermore, one may need to repeat these analyses as forecasts evolve to estimate the future financial protection benefits of vaccines. As a consequence, our results are related to the assumptions in these models such as the type of vaccines considered. Nevertheless, we pursued sensitivity analyses, and our results were robust to different sets of parameter assumptions that could have more importance on medical impoverishment and CHCs. Second, there are modelling assumptions from our financial protection simulation model itself with regard to the distributions of income and costs in the different populations. Some of our results, therefore, need to be interpreted with caution and also call for better data collection in the domain of OOP healthcare costs, access to healthcare and on risk factors to understand the distribution of cases across income quintiles. Furthermore, we presented a relatively simple FRP model, but one could develop more complexity that includes discounting and income growth over time (currently not incorporated in our model), as well as other social benefits or costs, for example. Another limitation is that we have chosen specific measures of FRP that rely on income-based thresholds to quantify the number of cases of CHC and poverty.^44^ This simplification also implies that the counterfactual excludes the possibility that these households would fall below the poverty line for other reasons during the same month. A final limitation of this analysis is the paucity of data concerning incidence of disease within a household.

Overall, our paper suggests that immunisation not only would have an important effect on economic well-being of a country but also would prevent disease-related hospitalisation, associated impoverishment and could provide significant financial protection to households, particularly the poorest who are at higher risks, have reduced access to healthcare and bear significant economic costs due to disease treatment. As the world aims to eliminate poverty by 2030, immunisation could play a key role in setting policy for the poverty reduction agenda.
