# Supplementary material for: Poverty reduction and equity benefits of introducing or scaling up measles, rotavirus and pneumococcal vaccines in low-income and middle-income countries: a modelling study
Source: BMJ Glob Health. 2018 Apr 9;3(2):e000613. doi: 10.1136/bmjgh-2017-000613 (PMC5898318; doi:10.1136/bmjgh-2017-000613)

Supplementary appendix. Poverty reduction and equity benefits of introducing or scaling up measles, rotavirus and pneumococcal vaccines in low- and middle-income countries: a modeling study by Riumallo Herl et al.

Table A 1 List of 41 Gavi-eligible low- and middle-income countries included in the study

Countries
Armenia
Bangladesh
Benin
Burkina Faso
Burundi
Cambodia
Cameroon
Chad
Comoros
Congo Republic
Côte d'Ivoire
Democratic Republic of the Congo
Ethiopia
Gambia
Ghana
Guinea
Haiti
Honduras
Indonesia
Kenya
Kyrgyzstan
Lesotho
Liberia
Malawi
Mali
Mozambique
Nepal
Niger
Nigeria
Pakistan
Rwanda
Senegal
Sierra Leone
Tajikistan
Tanzania
Timor-Leste
Togo
Uganda
Yemen
Zambia
Zimbabwe

Table A 2 Number of medical impoverishment cases in 1000's averted by vaccines in 41 low- and middle-income countries for those cohorts born between 2016 and 2030. 95% uncertainty ranges (UR) are given in parentheses.

	(1) Measles vaccine (95% UR)	(2) Pneumococcal conjugate vaccine (95% UR)	(3) Rotavirus vaccine (95% UR)
**Current coverage trends**			
Lowest	1,995 (1,718-2,285)	13 (6-21)	8 (3-16)
2nd Quintile	1,611 (1,348-1,860)	3 (0-6)	2 (0-4)
3rd Quintile	792 (628-946)	0 (0-0)	0 (0-0)
4th Quintile	582 (338-814)	0 (0-0)	0 (0-0)
Highest	0 (0-0)	0 (0-0)	0 (0-0)
Total	4,986 (4,486-5,466)	16 (9-24)	10 (4-18)
**Best case**			
Lowest	2,108 (1,812-2,415)	91 (70-114)	119 (93-145)
2nd Quintile	1,660 (1,384-1,912)	92 (72-112)	88 (66-113)
3rd Quintile	835 (664-1,006)	56 (40-71)	48 (34-65)
4th Quintile	618 (362-868)	29 (16-42)	16 (8-28)
Highest	0 (0-0)	0 (0-0)	0 (0-0)
Total	5,226 (4,694-5,738)	267 (232-307)	271 (230-314)

Note: Current coverage trends represent current vaccination forecasts. Best case includes additional Gavi funding for the expansion or implementation of vaccines. Figures present the average value of cases by economic quintile with 95% uncertainty ranges.

Table A 3 Percentage of medical impoverishment cases in 1000s averted by vaccines in 41 low- and middle-income countries for those cohorts born between 2016 and 2030. 95% uncertainty ranges (UR) are given in parentheses.

	(1)<br>Measles vaccine<br>(95% UR)	(2)<br>Pneumococcal conjugate vaccine (95% UR)	(3)<br>Rotavirus vaccine<br>(95% UR)
**Current coverage trends**			
Lowest	39.7% (35.2-44.3)	81.9% (60.8-98.0)	85.4% (55.0-100.0)
2nd Quintile	32.7% (28.3-37.1)	17.8% (2.0-9.2)	14.6% (0.0-45.0)
3rd Quintile	15.7% (12.7-18.7)	0.0% (0.0-0.0)	0.0% (0.0-0.0)
4th Quintile	11.8% (7.2-15.9)	0.0% (0.0-0.0)	0.0% (0.0-0.0)
Highest	0.0% (0.0-0.0)	0.0% (0.0-0.0)	0.0% (0.0-0.0)
**Best case**			
Lowest	40.1% (35.4-44.8)	34.2% (27.9-40.8)	43.8% (36.4-50.9)
2nd Quintile	32.1% (27.8-36.4)	34.2% (27.9-40.4)	32.5% (25.8-39.5)
3rd Quintile	15.9% (12.8-18.9)	20.8% (15.8-26.3)	17.7% (12.7-23.3)
4th Quintile	11.9% (7.3-16.1)	10.7% (6.3-15.5)	5.9% (2.8-9.8)
Highest	0.0% (0.0-0.0)	0.0% (0.0-0.0)	0.0% (0.0-0.0)

Note: Current coverage trends represent current vaccination forecasts. Best case includes additional Gavi funding for the expansion or implementation of vaccines. Figures present the average value of cases by economic quintile with 95% uncertainty ranges.

Table A 4 Total out-of-pocket (OOP) health expenditures (in 1,000,000 2011 International $) cases averted by vaccines in 41 low- and middle-income countries for those cohorts born between 2016 and 2030. 95% uncertainty ranges (UR) are given in parentheses.

	(1)<br>Measles vaccine<br>(95% UR)	(2)<br>Pneumococcal conjugate vaccine<br>(95% UR)	(3)<br>Rotavirus vaccine<br>(95% UR)
**Current coverage trends**			
Lowest	498 (421-571)	3 (2-5)	7 (5-10)
2nd Quintile	658 (551-756)	5 (4-7)	11 (7-15)
3rd Quintile	787 (658-904)	6 (4-8)	12 (7-15)
4th Quintile	979 (816-1,126)	9 (6-11)	18 (11-23)
Highest	1,283 (1,088-1,491)	11 (7-15)	20 (12-27)
Total	4,200 (3,910-4,517)	34 (29-40)	68 (56-79)
**Best case**			
Lowest	518 (439-592)	17 (15-20)	22 (18-25)
2nd Quintile	687 (577-787)	23 (20-27)	30 (25-35)
3rd Quintile	826 (692-948)	28 (24-32)	35 (29-40)
4th Quintile	1,026 (856-1,180)	36 (30-41)	44 (37-51)
Highest	1,357 (1,154-1,575)	55 (46-63)	59 (49-68)
Total	4,412 (4,111-4,736)	159 (147-170)	189 (175-203)

Note: Current coverage trends represent current vaccination forecasts. Best case includes additional Gavi funding for the expansion or implementation of vaccines. Figures present the average value of cases by economic quintile with 95% uncertainty ranges.

Table A 5 Percentage of total out-of-pocket (OOP) health expenditures averted by vaccines in 41 low- and middle-income countries for those cohorts born between 2016 and 2030. 95% uncertainty ranges (UR) are given in parentheses.

	(1)<br>Measles vaccine<br>(95% UR)	(2)<br>Pneumococcal conjugate vaccine (95% UR)	(3)<br>Rotavirus vaccine<br>(95% UR)
**Current coverage trends**			
Lowest	11.9% (10.1-13.7)	10.1% (6.5-14.0)	10.7% (6.8-14.9)
2nd Quintile	15.7% (13.4-18.0)	15.8% (10.6-21.2)	16.8% (10.9-22.3)
3rd Quintile	18.7% (16.0-21.4)	16.3% (11.0-22.4)	17.1% (10.9-22.7)
4th Quintile	23.3% (19.8-26.5)	24.9% (17.7-32.3)	26.3% (17.5-33.7)
Highest	30.4% (26.6-34.1)	32.9% (23.6-41.1)	29.3% (20.2-38.0)
**Best case**			
Lowest	11.8% (10.0-13.6)	11.0% (9.3-12.9)	11.7% (9.9-13.6)
2nd Quintile	15.7% (13.3-17.9)	14.7% (12.6-17.0)	15.9% (13.5-18.3)
3rd Quintile	18.7% (16.0-21.3)	17.7% (15.1-20.5)	18.3% (15.6-20.8)
4th Quintile	23.2% (19.8-26.4)	22.3% (19.3-25.3)	23.4% (19.9-26.5)
Highest	30.6% (26.8-34.3)	34.2% (30.1-38.0)	30.8% (26.8-34.3)

Note: Current coverage trends represent current vaccination forecasts. Best case includes additional Gavi funding for the expansion or implementation of vaccines. Figures present the average value of cases by economic quintile with 95% uncertainty ranges.

Figure A 1 Average coverage rate for each vaccine over 41 low- and middle-income countries over time for those cohorts born between 2000 and 2030

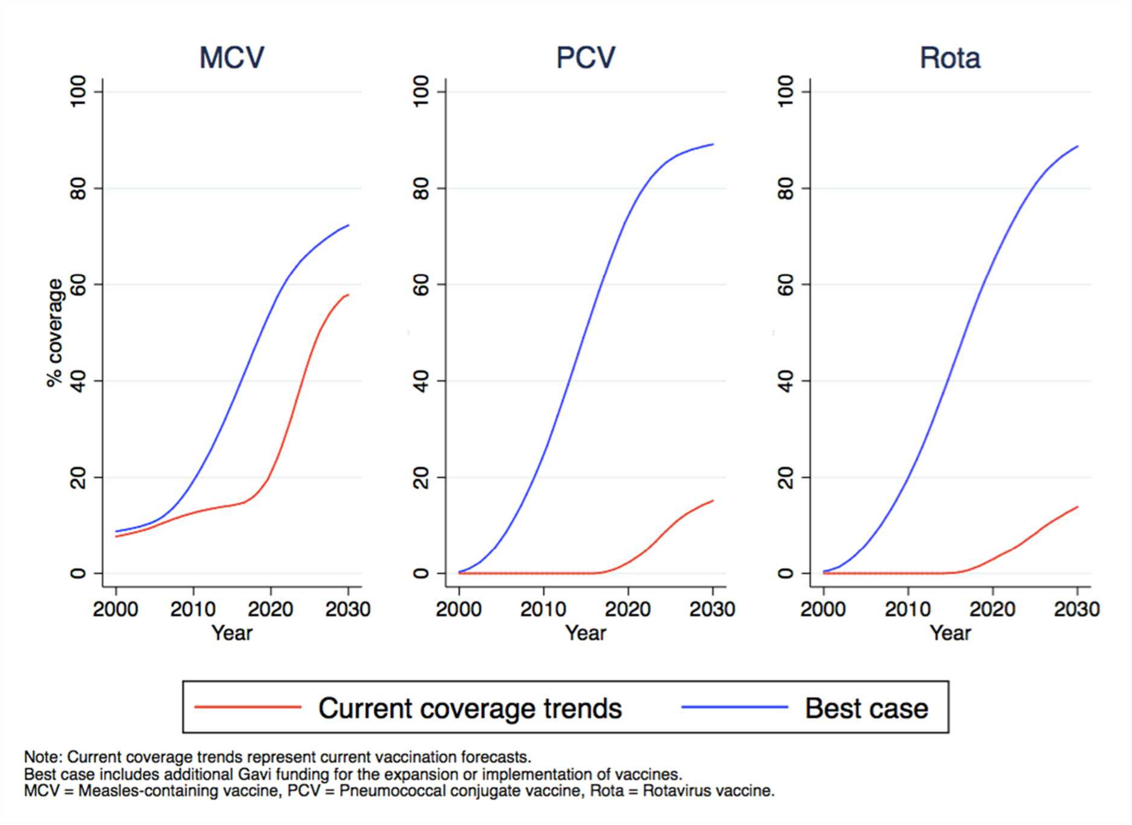

Figure A 2 Number of cases of catastrophic health costs averted by measles vaccine for each scenario under different assumptions of catastrophic thresholds (10%, 20%, 30%, and 40% of monthly household income) in 41 low- and middle-income countries for those cohorts born between 2016 and 2030

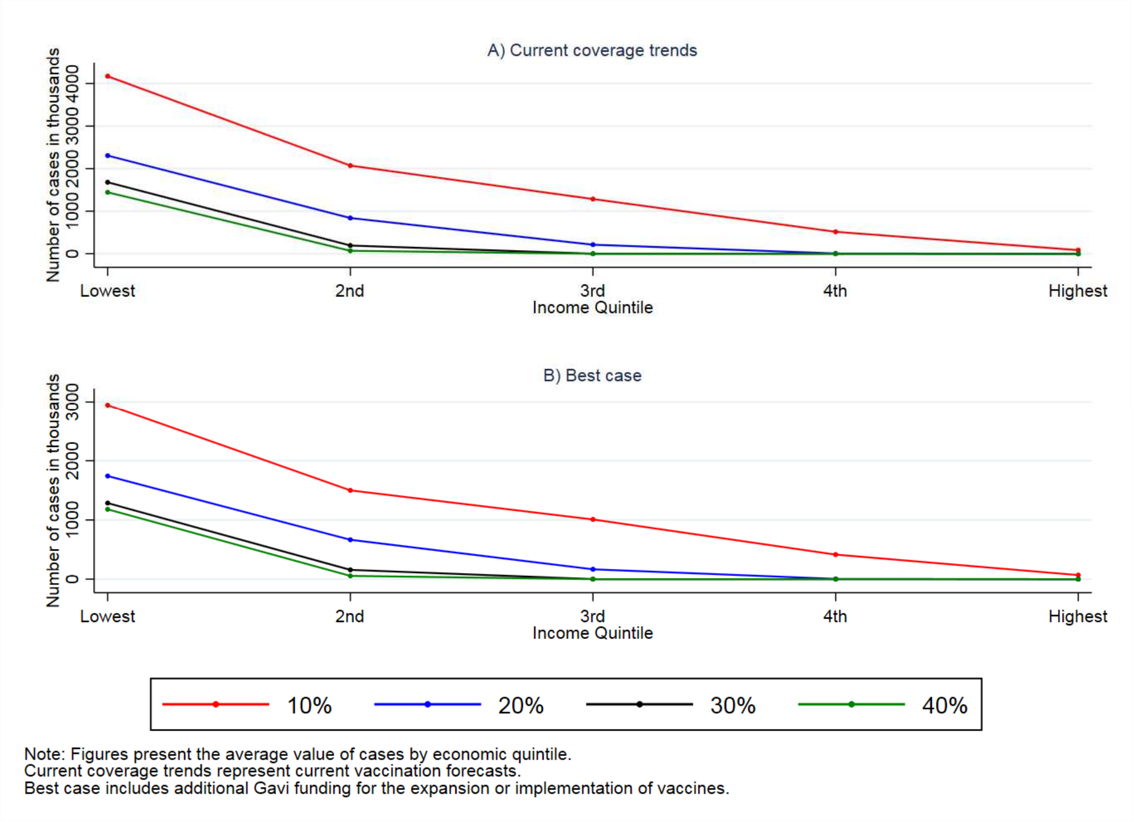

Figure A 3 Number of cases of catastrophic health costs averted by pneumococcal conjugate vaccine for each scenario under different assumptions of catastrophic thresholds (10%, 20%, 30%, and 40% of monthly household income) in 41 low- and middle-income countries for those cohorts born between 2016 and 2030

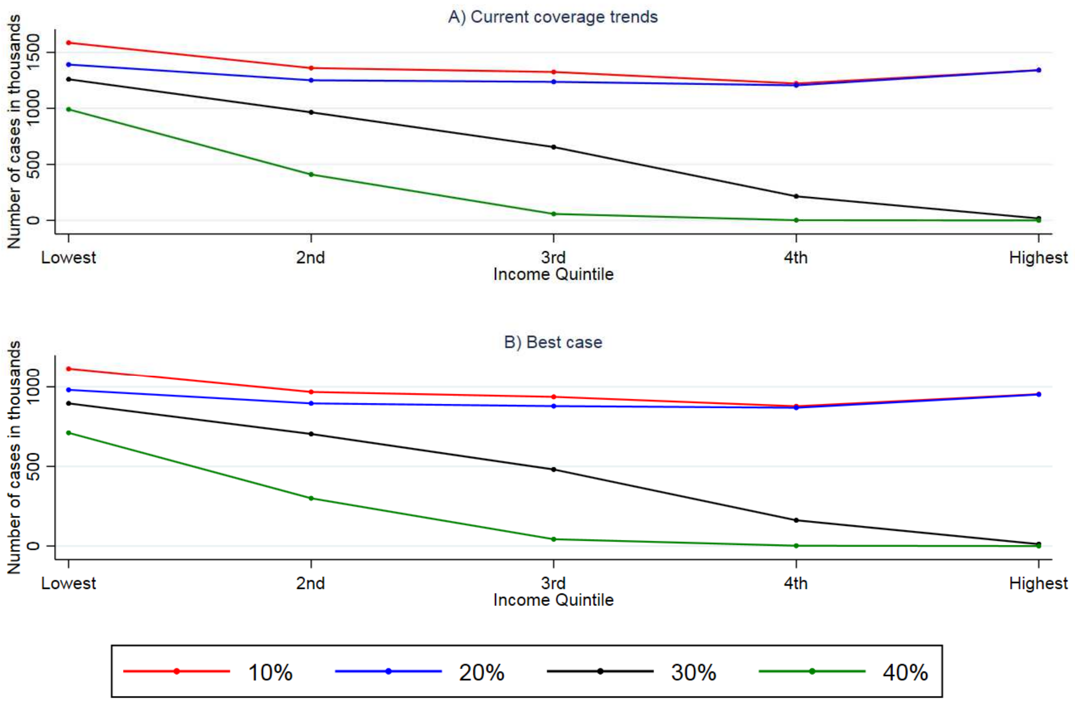

Note: Figures present the average value of cases by economic quintile.
Current coverage trends represent current vaccination forecasts.
Best case includes additional Gavi funding for the expansion or implementation of vaccines.

Figure A 4 Number of cases of catastrophic health costs averted by rotavirus vaccine for each scenario under different assumptions of catastrophic thresholds (10%, 20%, 30%, and 40%) in 41 low- and middle-income countries for those cohorts born between 2016 and 2030.

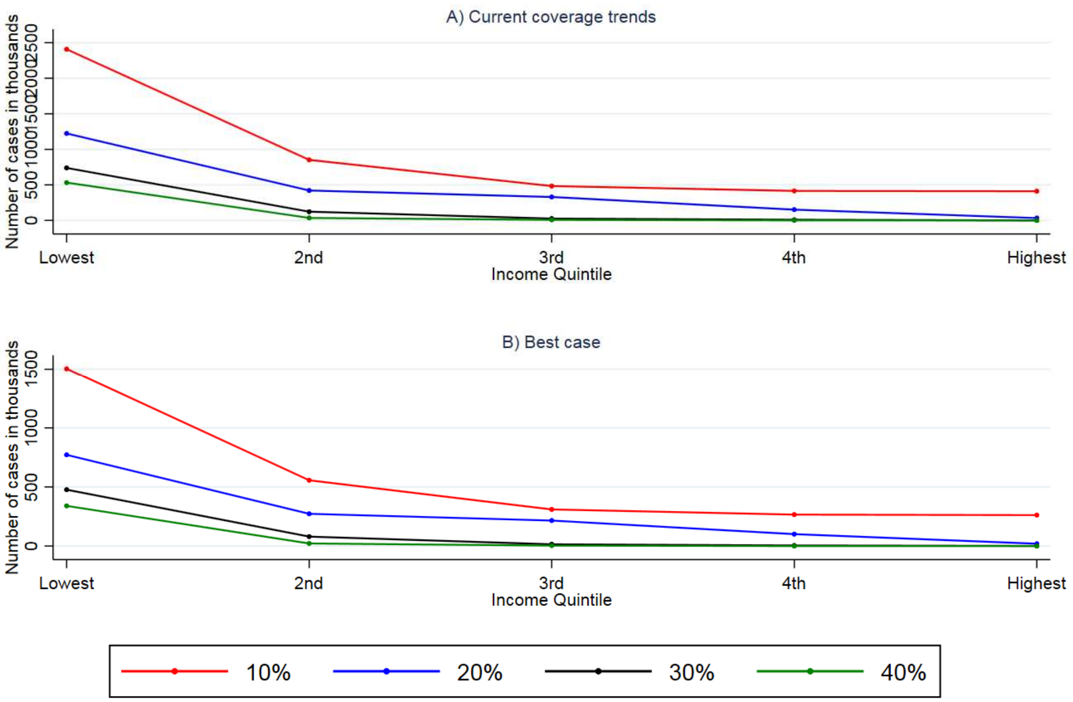

Note: Figures present the average value of cases by economic quintile.
Current coverage trends represent current vaccination forecasts.
Best case includes additional Gavi funding for the expansion or implementation of vaccines.

Figure A 5 Number of cases of catastrophic health costs averted by measles vaccine by scenario under assumption of largest health care utilization fraction by country in 41 low- and middle-income countries for those cohorts born between 2016 and 2030

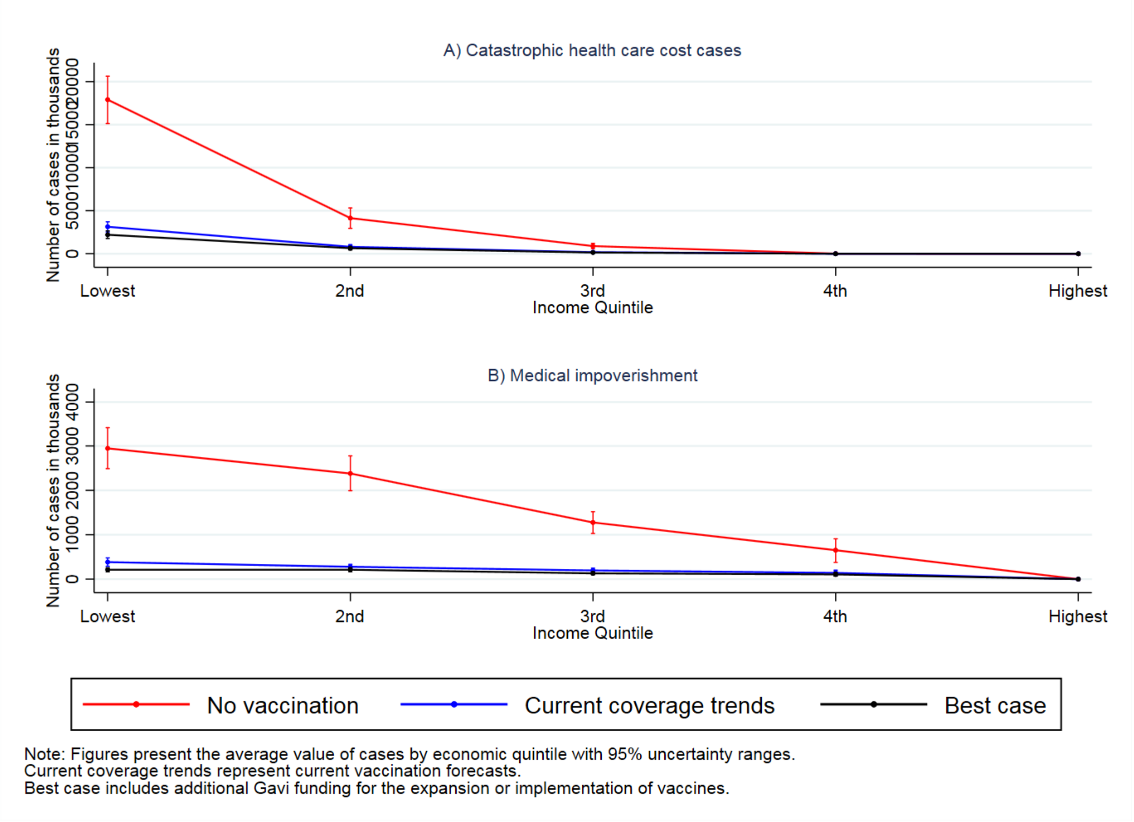

Figure A 6 Number of cases of catastrophic health costs averted by pneumococcal conjugate vaccine by scenario under assumption of largest health care utilization fraction (from highest quintile) by country in 41 low- and middle-income countries for those cohorts born between 2016 and 2030.

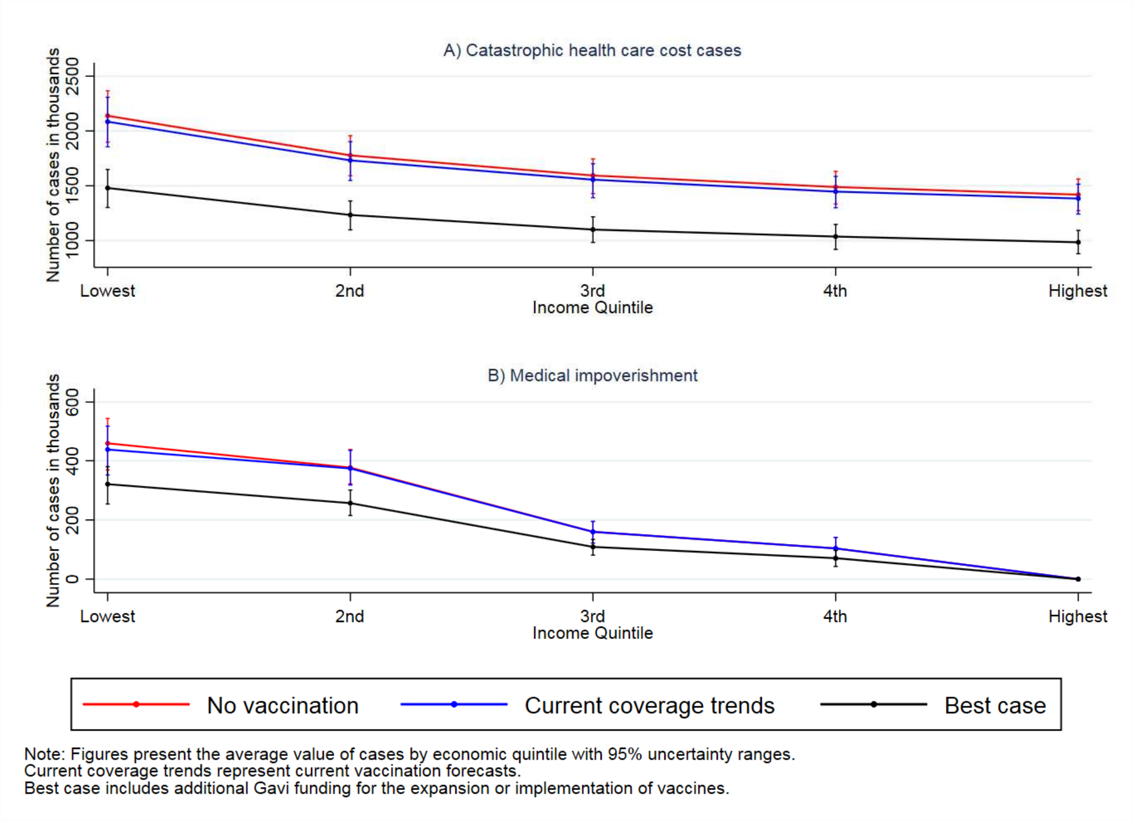

Figure A 7 Number of cases of catastrophic health costs averted by rotavirus vaccine by scenario under assumption of largest health care utilization fraction (from highest income quintile) by country in 41 low- and middle-income countries for those cohorts born between 2016 and 2030

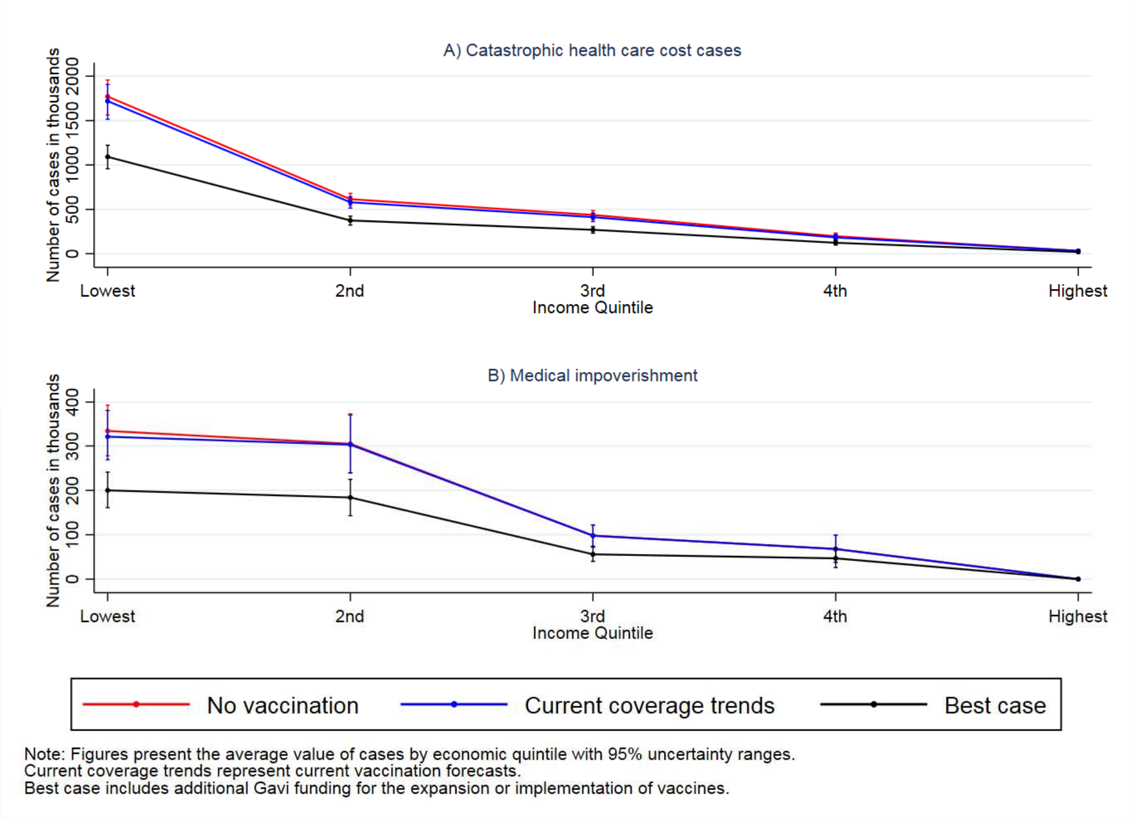

Figure A 8 Number of cases of catastrophic health costs averted by measles vaccine for each scenario under different assumptions of vaccine coverage with and without total fertility rate (TFR) adjustments in 41 low- and middle-income countries for those cohorts born between 2016 and 2030.

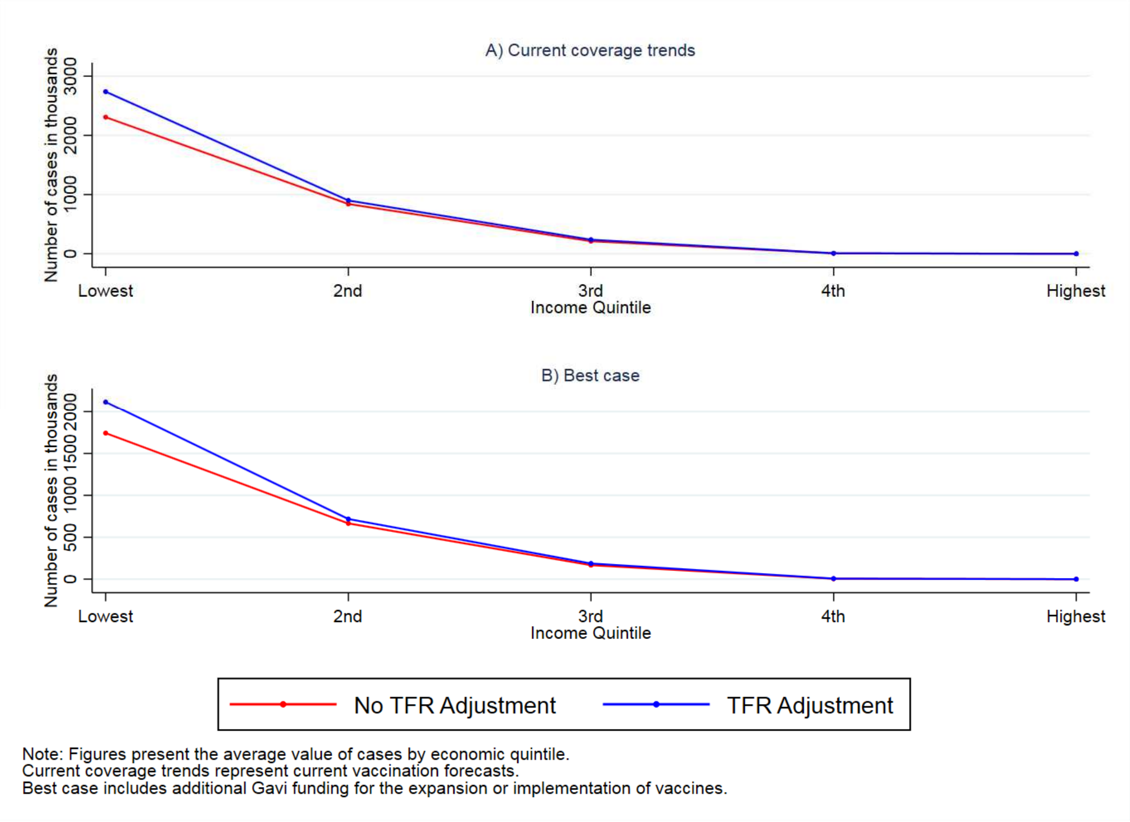

Figure A 9 Number of cases of catastrophic health costs averted by pneumococcal vaccine for each scenario under different assumptions of vaccine coverage with and without TFR adjustments in 41 low- and middle-income countries for those cohorts born between 2016 and 2030.

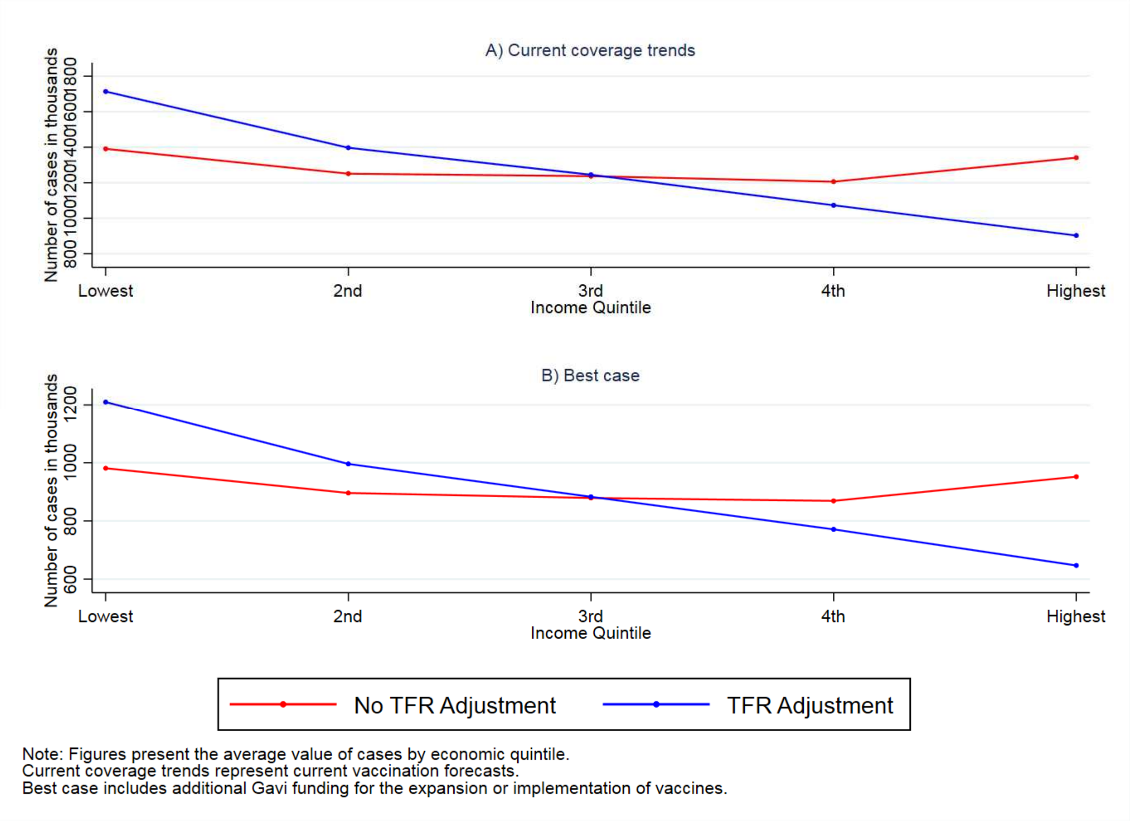

Figure A 10 Number of cases of catastrophic health costs averted by rotavirus vaccine for each scenario under different assumptions of vaccine coverage with and without TFR adjustments in 41 low- and middle-income countries for those cohorts born between 2016 and 2030.

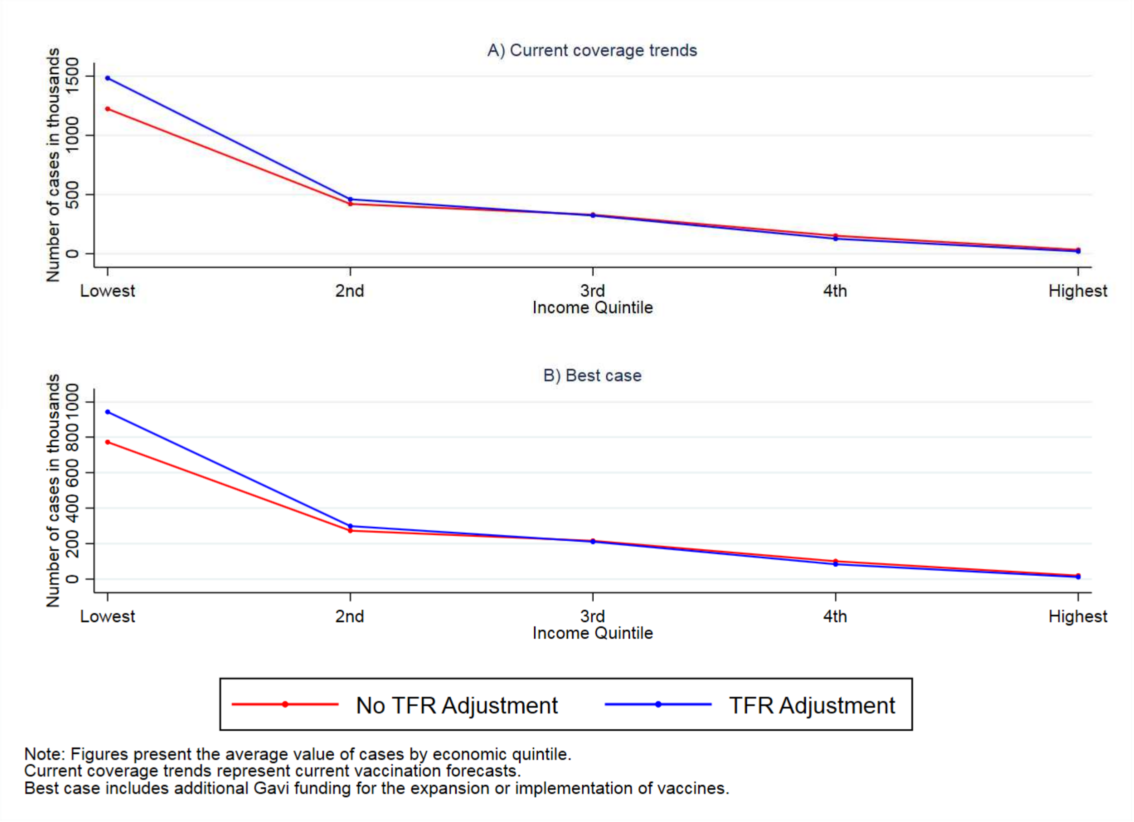

Supplement: Supplementary file 1 [file bmjgh-2017-000613supp001.pdf]
